# Mr. Davies's Case of Incipient Mortification

**Published:** 1801-06

**Authors:** H. Davies

**Affiliations:** Piccadilly


### *To the Editors of the Medical and Phyſical Journal.*

Gentlemen,

If the following caſe of an incipient mortification of the fingers, from immerſing the hand in cold water, together with the plan of treatment adopted, be thought of ſufficient importance to occupy a page or two of your uſeful Journal, you will add to the obligations already conferred by your polite attention to ſome former communications, in permitting it to appear in your next.

I am, &c.

*Piccadilly, May 6, 1801.* H. DAVIES.

---

A woman about forty years of age, of a thin, ſpare habit of body, in conſequence of immerſing her hands in cold water, on the next day felt an unuſual pain in the arm, from the hand up to the elbow. An inflammation enſued, which very ſoon put on a dark, livid appearance, attended with a conſiderable increaſe of pain, on the following day, which laſted for three hours together without any interval of eaſe. This continued for three days before ſhe conſulted me on the caſe. On the fourth day I was called to attend her, when I found the fingers and thumb exhibiting a mortified appearance. The ſenſibility and mobility of the parts were nearly deſtroyed; the pulſe weak, attended by a general debility of the whole ſyſtem, which cauſed ſome apprehenſion leſt a complete gangrene of the parts affected might conſtitute the unhappy termination.

Under theſe circumſtances, I thought the indication was plain and ſimple; that is, to reſtore, as much as poſſible, energy to the ſyſtem in general, vitality to the affected parts in particular, and, likewiſe, to diminiſh the morbid irritability neceſſarily attendant tendant on fuch cafes ; confequently, I exhibited the cinchona in as concentrated a form as I could, and to be taken in as full a dofe, and as often as the ftomach could bear, with the addition of tinct. opii, together with an anodyne at night. The parts were ordered to be fomented often in the day with equal parts of brandy and vinegar, and then covered with linen rags, wetted with camphorated fpirits. A generous diet was enjoined, and a liberal ufe of wine enforced. I was happy to find, on the adoption of this plan, fome promifing appearances of amendment on the next day. Senfibility, in fome meafure, was reftored to the parts, and the difcoloured fkin affumed a more pleafing afpect. Perfifting in the fame plan, appearances every day became more favourable. The pain through the whole fubftance of the arm gradually diminifhed, and the whole fyftem, before enervated, daily increafed in vigour.

From fuch appearances little doubt was to be entertained refpecting the iffue of a cafe, which, without fuch timely applications, portended the moft ferious confequences. Not that I would be underftood to claim the merit of the difcovery of any new remedy employed in the treatment of it, but it is rather written in confirmation of one already well known and underftood, even bark and opium, the efficacy of which, in counteracting gangrenous tendency, has been repeatedly and advantageoufly experienced.

The late Mr. Pott, in treating of a mortification of the toes and feet, obferved, that "Among the many cafes in which the virtues of the Peruvian bark are particularly and juftly celebrated, muft be reckoned the diftempers called gangrene and mortification. Its general efficacy in ftopping one and refifting the other, have made no inconfiderable addition to the fuccefs of chirurgic art." And, as it acquires no inconfiderable improvement by its junction with opium, in cafes of that defcription, it cannot, perhaps, be too early employed or freely exhibited in the curative plan of treatment.

If this fhort narrative of the fuccefsful termination of an unpromifing cafe, ferves to direct the practice of the lefs experienced in the profeffion, or tends to eftablifh the reputation of an approved remedy in the hands of the judicious, fo as in any degree to promote the intereft of the healing art, the writer will be amply recompenfed for the little time he has beftowed in collecting the few particulars together.

